# Alveolar wars: The rise of in vitro models to understand human lung alveolar maintenance, regeneration, and disease

**DOI:** 10.1002/sctm.19-0433

**Published:** 2020-04-09

**Authors:** Kelly V. Evans, Joo‐Hyeon Lee

**Affiliations:** ^1^ Wellcome – MRC Cambridge Stem Cell Institute, Jeffrey Cheah Biomedical Centre University of Cambridge Cambridge UK; ^2^ Department of Physiology, Development and Neuroscience University of Cambridge Cambridge UK

**Keywords:** alveolar organoids, human lung disease, in vitro models, lung regeneration, lung stem cells

## Abstract

Diseases such as idiopathic pulmonary fibrosis, chronic obstructive pulmonary disease, and bronchopulmonary dysplasia injure the gas‐exchanging alveoli of the human lung. Animal studies have indicated that dysregulation of alveolar cells, including alveolar type II stem/progenitor cells, is implicated in disease pathogenesis. Due to mouse‐human differences, there has been a desperate need to develop human‐relevant lung models that can more closely recapitulate the human lung during homeostasis, injury repair, and disease. Here we discuss how current single‐cell RNA sequencing studies have increased knowledge of the cellular and molecular composition of human lung alveoli, including the identification of molecular heterogeneity, cellular diversity, and previously unknown cell types, some of which arise specifically during disease. For functional analysis of alveolar cells, in vitro human alveolar organoids established from human pluripotent stem cells, embryonic progenitors, and adult tissue from both healthy and diseased lungs have modeled aspects of the cellular and molecular features of alveolar epithelium. Drawbacks of such systems are highlighted, along with possible solutions. Organoid‐on‐a‐chip and ex vivo systems including precision‐cut lung slices can complement organoid studies by providing further cellular and structural complexity of lung tissues, and have been shown to be invaluable models of human lung disease, while the production of acellular and synthetic scaffolds hold promise in lung transplant efforts. Further improvements to such systems will increase understanding of the underlying biology of human alveolar stem/progenitor cells, and could lead to future therapeutic or pharmacological intervention in patients suffering from end‐stage lung diseases.

## Graphical abstract

This review highlights the current concepts and advances in the research fields of human lung alveoli. Combination of in vitro human lung alveolar model systems with single‐cell technologies of “omics” approaches will improve our understanding of normal human lung development, maintenance and regeneration, paving the way for future therapeutic repair of diseased lungs.

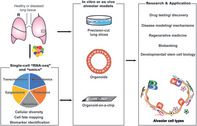

**Significance statement**

Over the last decade, stem cell‐derived culture model systems of human lungs have garnered renewed interest, as they recapitulate human lung tissues in a dish. This study summarizes the current concepts and advances in the field of human distal lung alveoli, which is the most critical region for the respiratory function and disease, and thereby has been moving forward so rapidly. Specifically, this study compares the differences in cellular compositions of distal lungs between mouse and human and discusses the current model systems to study maintenance, regeneration, and disease of human lung alveoli, which is difficult to model in animal studies.

## 1 INTRODUCTION

The primary function of the lungs is gas exchange and the site for this is the alveoli that are arranged by acini found in the lung parenchyma regions. There is a significant need to understand the mechanisms of alveolar maintenance and damage repair because damage to the alveolar region is a component of chronic adult lung diseases such as chronic obstructive pulmonary disease (COPD) and idiopathic pulmonary fibrosis (IPF) and a cause of acute respiratory failure in pneumonia and acute respiratory distress syndrome (ARDS). In addition, insufficient generation of alveoli results in various neonatal and infant diseases including bronchopulmonary dysplasia (BPD). Despite the pivotal role of alveoli in lung function and disease, and their clinical burden, the pathogenesis of these diverse diseases is incompletely understood and treatment options for patients remain limited. This is partly due to the lack of model systems that allow us to understand human lung biology and disease.

**FIGURE 1:**
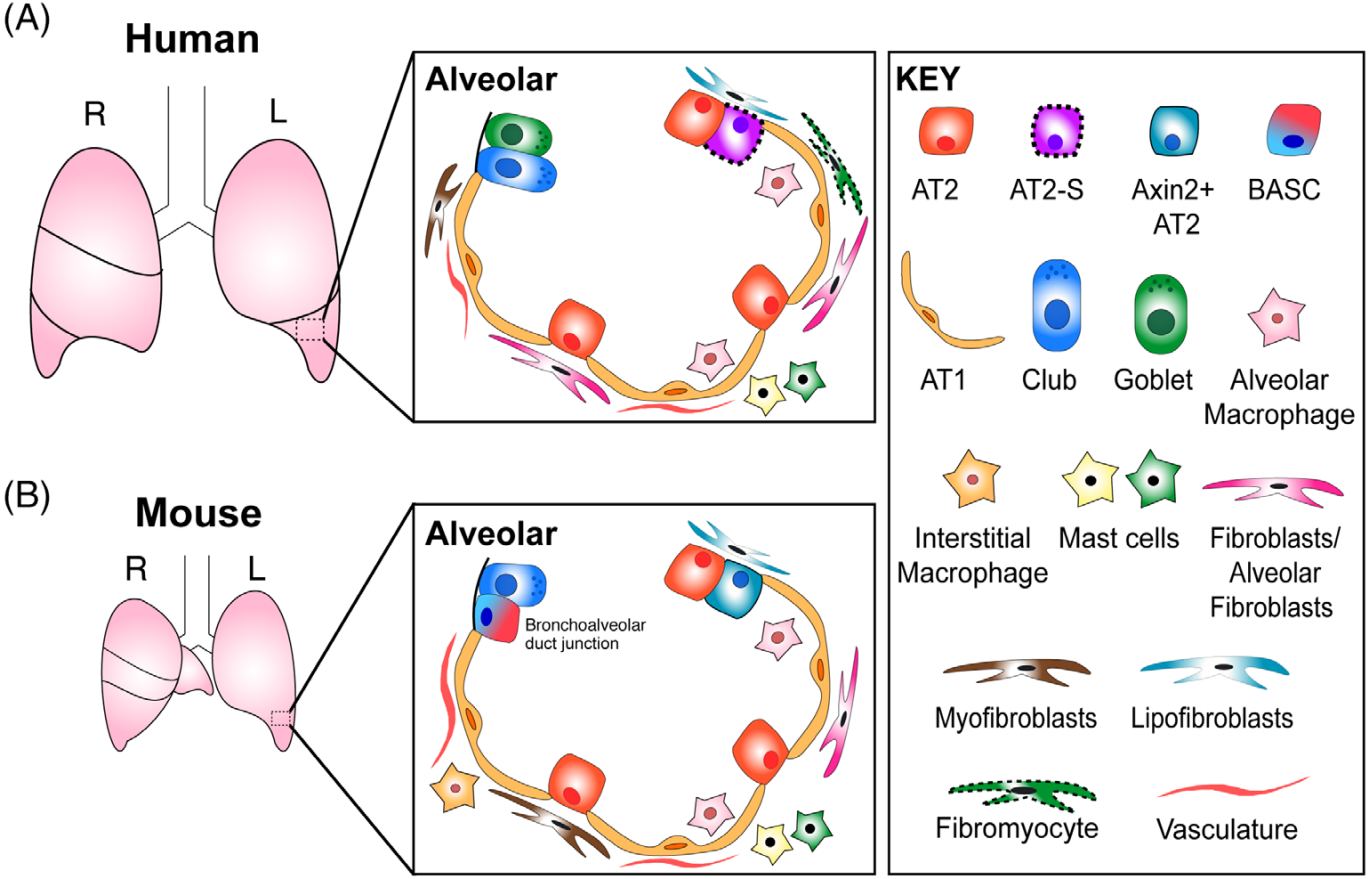
Cellular composition of the mouse and human lung. A, Schematic of the adult human lung alveoli. The adult human lung is split into five lobes; three on the right, and two on the left. The distal alveolar region has two main epithelial cell types; surfactant‐producing AT2 cells and gas‐exchanging AT1 cells. A subtype of AT2 cells, named AT2‐signaling or AT2‐s, has been suggested to show an enrichment of Wnt pathway genes from scRNA‐seq analysis, although their presence needs to be verified.^1^ Alveolar macrophages exist within the alveolar space, while two populations of mast cells have recently been identified. Fibroblast heterogeneity also exists, with lipofibroblasts, myofibroblasts, and recently identified alveolar fibroblasts located in the alveoli. Cells with dotted outlines have not yet been fully verified. B, Schematic of the adult mouse lung. The mouse lung is also split into five lobes; four on the right, and one on the left. The mouse distal alveolar region possesses at least two subsets of AT2 cells, with AT2 cells expressing *Axin2* (*Axin2* ^*+*^ AT2) having increased stem cell activity.^2^, ^3^ The bronchoalveolar duct junction is an area of transitional epithelium between the alveoli and distal bronchioles, and contain bronchoalveolar stem cells (BASCs); a cell type that expresses both Sftpc and Scgb1a1, and have been shown to differentiate to alveolar and bronchiolar lineages following bleomycin‐ and naphthalene‐induced lung damage, respectively.^4^, ^5^ Such a region does not exist in the human lung. Furthermore, basal cells, although present in the human distal lung, are restricted to the trachea and mainstem bronchi of the mouse lung

In this review, we summarize our current knowledge of human lung alveoli from decades of animal studies and recent single‐cell RNA sequencing analysis (scRNA‐seq) (Figure 1). We also highlight recent advances in the available in vitro and ex vivo human lung alveolar model systems and discuss their potential applications and limitations in therapeutic aspects.

### 1.1 Structure and composition of human lung alveoli

**FIGURE 2:**
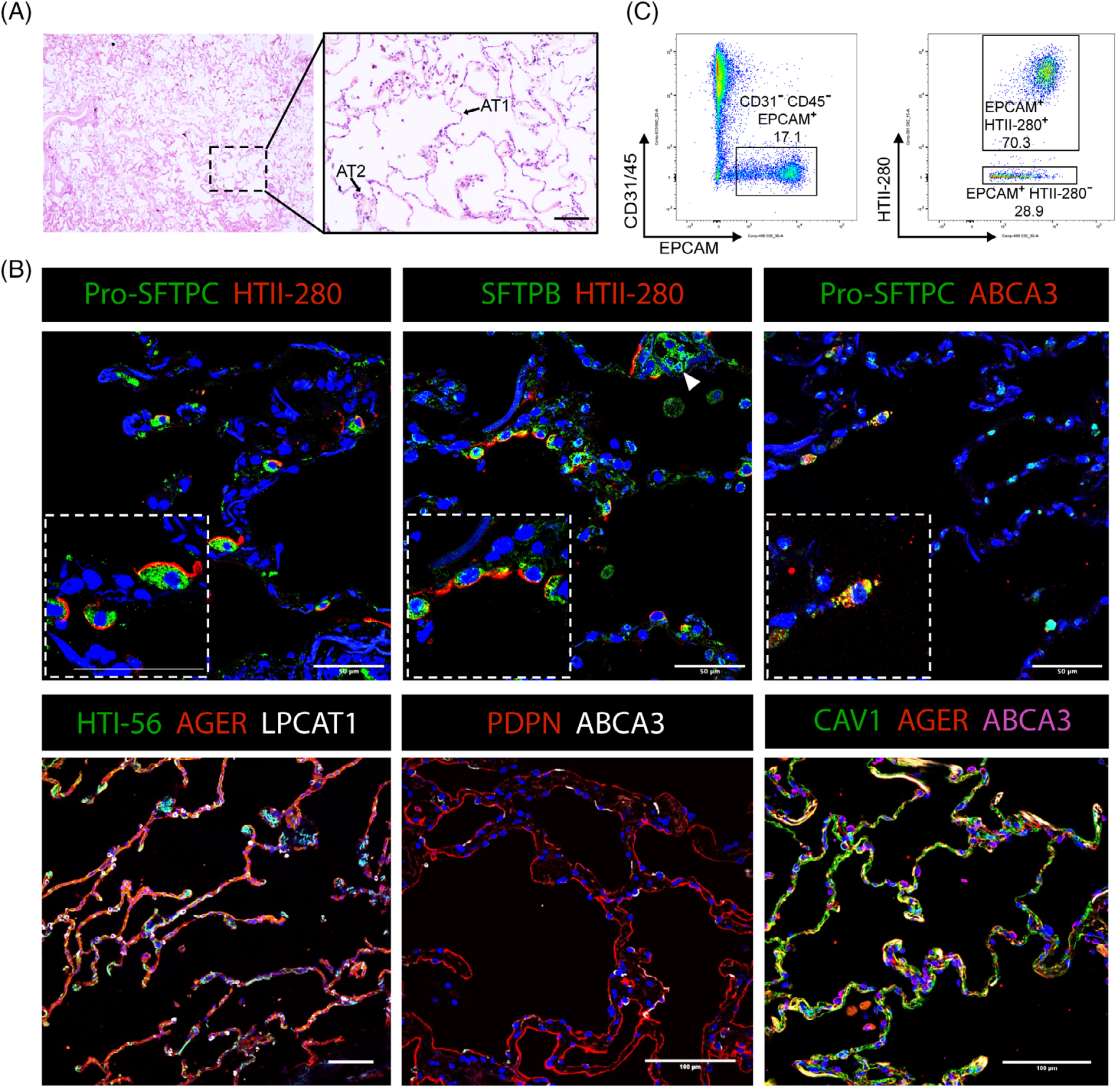
Composition and cellular markers of the healthy human distal lung. A, Representative hematoxylin and eosin (H&E) staining of the healthy human adult distal lung tissue shows open alveolar spaces and thin alveolar walls, with the presence of AT1 and AT2 cells. Scale bar = 100 μm. B, Representative immunofluorescence (IF) staining of the healthy human adult distal lung tissue sections for canonical AT2 marker genes including pro‐SFTPC (green, top left and top right), HTII‐280 (red, top left and middle), SFTPB (green, top middle), ABCA3 (red, top right; white, bottom middle; pink, bottom right), and LPCAT1 (white, bottom left) and AT1 marker genes including PDPN (red, bottom middle), HTI‐56 (green, bottom left), AGER (red, bottom left and right), and CAV1 (green, bottom right). Of note, some AT2 cells do not express HTII‐280 (Arrowhead: SFTPB^+^ HTII‐280^−^ cell cluster). Scale bar = 50 μm unless stated otherwise. C, Flow‐cytometry analysis cell sorting (FACS) plot of primary human lung cells isolated from a normal background parenchyma lung donor following mechanical and enzymatic tissue dissociation. Cells were analyzed for CD31‐APC, CD45‐APC, EPCAM‐FITC, and HTII‐280‐PE. AT2 cells represent CD31^−^CD45^−^EPCAM^+^HTII‐280^+^ populations, where they consistently represent more than 70% of EPCAM^+^ cells in the distal parenchyma lung tissues. Normal human background tissue was obtained from deidentified lungs of adult donors that were deemed unsuitable for transplantation

The alveoli are made up of two types of epithelial cells, macrophages, vascular and mesenchymal cells (Figure 1). Alveolar type I cells (AT1) are thin squamous epithelial cells allowing oxygen diffusion into underlying capillaries and cover ~96% of the lung surface area (Figure 2A,B). Alveolar type II cells (AT2) are cuboidal epithelial cells found in the alveolar corner and produce surfactant—a mixture of lipids and proteins, which maintain low alveolar surface tension, preventing the delicate structure of the alveolar sacs from collapsing upon breathing (Figure 2A,B).^6^, ^7^, ^8^ AT2 cells also have functions in immune response by having the ability to respond to innate immune stimuli.^9^ During development both AT1 and AT2 cells are derived from common multipotent alveolar progenitor cells in the canalicular‐saccular phases of human lung development (16‐36 postconception weeks), although there is no evidence whether such cells exist in the mature lung.^10^, ^11^ The maintenance and regeneration capacity of an adult alveolar epithelium is defined by the presence of AT2 cells which behave as facultative stem cells, with both traditional two‐dimensional (2D)‐cultures of human AT2 cells and later 3D lung organoid studies indicating that AT2 cells can self‐renew and differentiate into AT1 cells.^8^, ^9^, ^12^, ^13^ Recent work has suggested that there may be an underappreciated heterogeneity in the lung, including within the AT2 cell population (Figure 1). TM4SF1^*+*^ AT2 cells have been suggested to possess better capacity to proliferate and produce AT1 cells when necessary, with increased responsiveness to Wnt signaling demonstrated in human AT2 cell‐derived organoid culture.^2^ A recent scRNA‐seq analysis of selectively enriched epithelial populations from whole human donor lungs also supported the potential heterogeneity of AT2 cells by showing a distinct cluster of AT2 cells, named AT2‐signaling, expressing Wnt pathway genes.^1^ Additional studies have not reported such AT2 cell subpopulations in their scRNA‐seq analysis of whole human lung cells, which may be due to differences in sequencing platforms and cell preparation.^14^ However, further validation and phenotypic analysis of these populations is required to understand their functional distinction, if any, in lung maintenance and regeneration. It still remains to be answered: (a) Are certain subpopulations more potent, perhaps having increased capacity for regeneration? (b) Or, do broad AT2 cells have plasticity to be activated upon damage? (c) What are the signals inducing this heterogeneity? (d) Are specific subsets more prone to become damaged during disease progression? Furthermore, work in the mouse has revealed that airway cells including club cells, bronchioalveolar stem cells (BASCs), and clusters of cells expressing Krt5 contribute to alveolar cells following severe damages, highlighting injury‐induced cellular plasticity, but it is currently unknown whether this can also occur in the human lung.^4^, ^5^, ^15^, ^16^, ^17^, ^18^, ^19^ Due to the inability to perform in vivo studies of cells that reside in the human lung alveoli, the establishment of in vitro models such as human lung organoids, and ex vivo cultures such as precision‐cut lung slices (PCLS) have been successful in modeling aspects of the human lung alveoli, and were described in further detail later in this review. For example, one such study reported that human AT1 cells can de‐differentiate to AT2‐like cells in in vitro culture.^20^

The lung mesenchyme is an important source of morphogenetic and specification signals, and gives rise to cells including smooth muscle, fibroblasts, and the endothelium (Figure 1). However, little is known about the cellular diversity and mechanisms of their maintenance, in part due to a lack of defined markers. Traditionally, fibroblasts in the alveoli have been characterized as alveolar fibroblasts and lipofibroblasts, although their exact roles remain to be defined, and there has even been some controversy regarding the presence of lipofibroblasts in the human lung.^21^, ^22^ Regional fibroblast heterogeneity has been suggested in the human lung.^23^, ^24^ Recent advances in scRNA‐seq analysis have begun to prise apart subsets of mesenchymal cells, with multiple distinct stromal cell populations being identified in adult human lungs, including muscle cells, pericytes, and multiple fibroblast populations. *COL1A1* ^+^ fibroblasts can be split into two subpopulations according to their gene expression profiles gained from scRNA‐seq analysis; alveolar fibroblasts, and adventitial fibroblasts localized to vascular adventitia, while two *ACTA2*‐enriched populations comprising myofibroblasts and previously unseen fibromyocytes that exhibit high expression of contractile genes have been observed within proximal lung tissue (Figure 1A).^1^ These cells await further characterization, validation and identification of exact spatial location within the lung. scRNA‐seq reported enrichment of Hh target genes in the proximal mesenchyme compared to distal mesenchyme.^25^ Differences in contractile forces have also been reported.^26^ Such regional differences in stromal populations may partially explain the varied effects of injury and repair in distinct areas of the lung for different respiratory diseases. Unexpected molecular diversity has also been discovered in the endothelium, the cells of which play an important role in vascular homeostasis and allow for efficient gas exchange in the lungs, including two molecularly distinct capillary cell populations located in human alveoli.^1^ With the recent identification of these further subtypes, phenotypic assessment of these cells remains to be elucidated. Utilizing in vitro cocultures, the population of mesenchymal cells can be cocultured with other cell types including epithelial cells, which can affect their growth or differentiation abilities, providing a beneficial platform for which to determine the roles of individual mesenchymal populations and their crosstalk.^27^ Using surface marker information from recent scRNA‐seq data sets, novel populations of mesenchymal cells could be isolated and cultured, allowing for their validation and phenotypic analysis.

Immune cells such as macrophages are also important cellular components of the human lung alveoli. Alveolar macrophages (AMs) are located on the luminal epithelial surface of the alveoli, making up more than 95% of phagocytes in the alveoli at steady state, and act as the first line of defense against invading pathogens and clear the surfactant.^28^, ^29^, ^30^ scRNA‐seq data have identified proliferating and nonproliferating AMs in healthy human lungs, while AM heterogeneity has been observed in disease settings such as IPF, where a subset of monocyte‐derived AMs express profibrotic genes.^1^, ^14^, ^31^ How such populations affect other alveolar cell populations, and their contribution to disease states is currently unknown, and await phenotypic assessment. Understanding of immune cells within human lungs is important, as many acute and chronic lung diseases are associated with an increase in inflammation.

### 1.2 Cellular dysfunction in lung diseases

**FIGURE 3:**
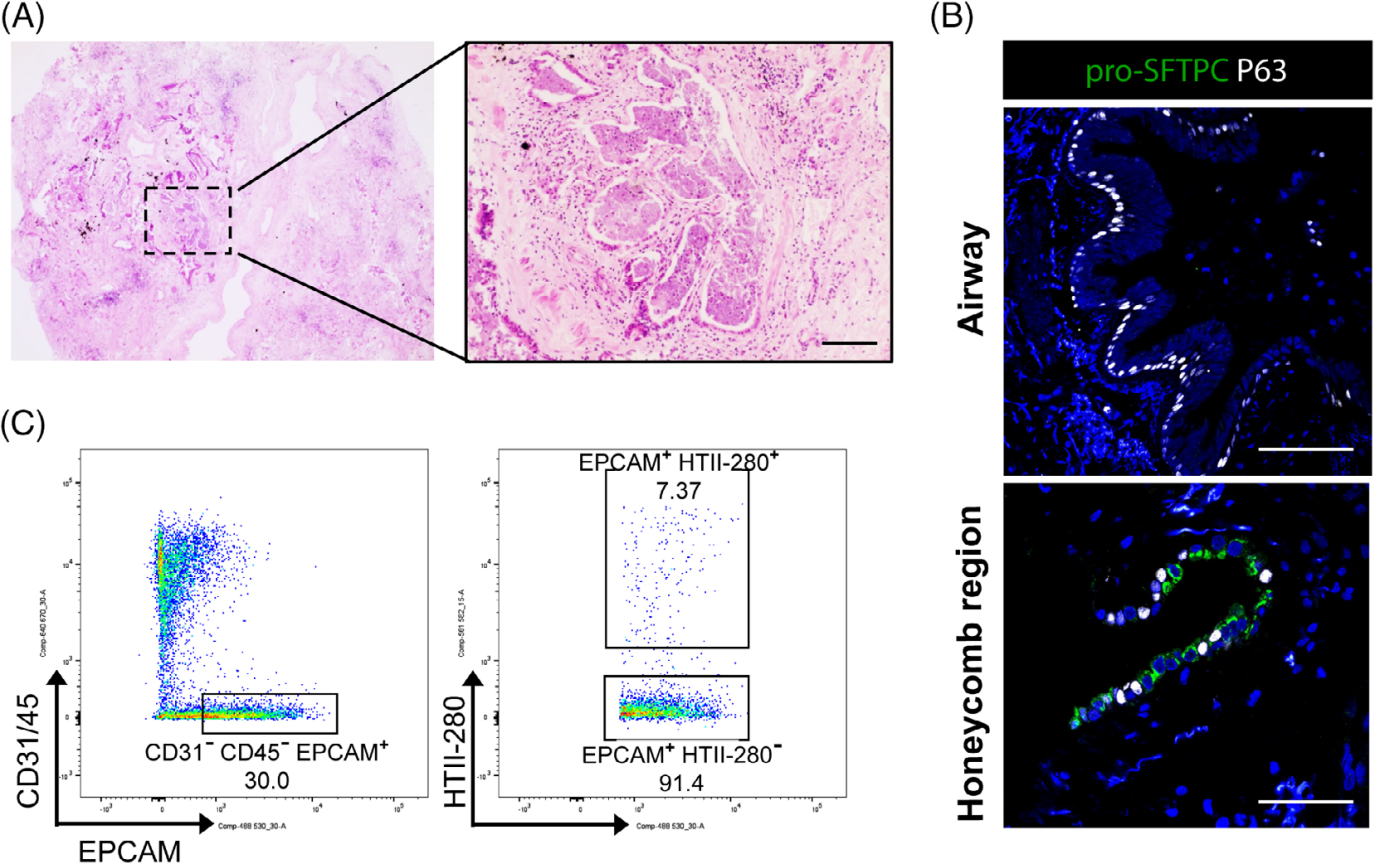
Composition and cellular markers of IPF human distal lung. A, Representative H&E image of distal lung parenchyma tissue sections from IPF patients. Alveolar architecture is completely destroyed, with the presence of fibroblastic foci and honeycomb regions (inset). Scale bar = 100 μm. B, Representative IF stainings of distal lung tissue sections from IPF patients for canonical airway and alveolar lineage markers. Normal airway structures still exist in IPF lung tissue, with basal cells expressing P63 (white, top panel) but none of AT2 markers such as pro‐SFTPC (green, top panel) Of note, aberrant cell types exist within honeycomb regions of IPF lung tissue, with cells expressing airway (P63) and AT2 markers (pro‐SFTPC) existing in close proximity of each other (bottom panel). Scale bar = 50 μm. C, FACS plot of primary human lung cells isolated from lung parenchyma tissues of IPF patients following mechanical and enzymatic tissue dissociation. Cells were analyzed for CD31‐APC, CD45‐APC, EPCAM‐FITC, and HTII‐280‐PE. Of note, the number of EPCAM^+^HTII‐280^+^ cells is dramatically reduced in IPF lung tissues vs healthy adult human parenchyma lung tissues. Human IPF tissue was obtained from deidentified lungs of adult donors at the time of transplantation

Dysregulation of cellular homeostasis or lack of alveolar structures has been implicated in lung diseases. IPF is an interstitial lung disease characterized by honeycomb lesions, hyperplastic AT2 cells, and fibroblastic foci, the “active lesions” of the disease (Figure 3A). Prognosis is poor and current treatments are limited, with only two drugs, Pirfenidone and Nintedanib, that possess antifibrotic and antiinflammatory properties, available in the clinic.^32^, ^33^, ^34^, ^35^ Although shown to provide a survival advantage to some patients, prevention or reversal of fibrosis has not been achieved, and disease progression is inevitable.^32^, ^33^, ^35^ Repeated injury to AT2 cells, possibly in a genetically sensitive background, has been suggested to lead to disease.^36^ Additionally, evidence indicating that some familial and sporadic forms of IPF comprise mutations within AT2 cells, such as within the SFTPC gene, provides further support.^37^, ^38^, ^39^ Furthermore, telomere shortening within AT2 cells has also been implicated in the disease.^40^ One of the key features in IPF is “bronchiolization” of the distal lung tissue showing an increase in the number of airway cells, and coexpression or close localization of cells expressing alveolar and airway transcripts (Figure 3B).^14^, ^41^ This observation is supported by recent scRNA‐seq studies describing unique populations of *SOX2* ^*−*^ *SOX9* ^*+*^ *KRT5* ^*−*^ *KRT17* ^*+*^ cells (aberrant basaloid cells) by Adams et al and *KRT5* ^*−*^ *KRT17* ^*+*^ cells by Habermann et al in human IPF peripheral lung tissue.^31^, ^42^ These cells may be implicated in IPF pathogenesis due to both their proximity to myofibroblast foci and expression of genes such as pathological extracellular matrix (ECM), epithelial‐to‐mesenchymal (EMT)‐related markers, and senescence‐associated genes, although characterization and phenotypic analysis of these populations needs to be performed in order to identify and validate their cellular identity and activity.^31^, ^42^ Could they be derived from alveolar or airway progenitors, or perhaps a subpopulation of a so far unidentified progenitor population? Are they responding to signals from a dysregulated microenvironment, or perhaps failing to respond to the correct signals due to an intrinsic dysfunction? It would be interesting to ask whether the emergence of this aberrant cell population is reversible and their functional implications in the pathogenesis of IPF. Due to the suggested role of AT2 cells in IPF, it is important to better understand this population to gain improved insights into disease initiation and progression. Animal models do not fully recapitulate the pathogenesis of human IPF, due to issues such as reliance on exogenous factors for injury initiation and resolution of fibrosis.^38^ Therefore, better understanding of human lung alveolar cells is also required to complement animal studies. in vitro models such as lung organoids may prove useful in answering such questions, particularly whether regenerative capacity of AT2 cells in IPF is disrupted, and if so, how to revert their repopulating potential. IL‐13 has been implicated in IPF pathogenesis by upregulation in bronchoalveolar lavage fluid of IPF patients.^43^ Treatment of human AT2‐derived alveolar organoids with IL‐13 leads to a reduction of SFTPC^+^ cells. As these organoids were cocultured with supporting stromal cells, it is currently unknown whether the effect of IL‐13 is direct, or whether it is acting on the stromal cells. Myofibroblasts are considered to be vital contributors to fibrotic diseases, and activation of fibroblasts to myofibroblasts is thought to be driven by TGFβ signaling, but the exact cellular source remains unknown. A *HAS1* ^*hi*^ ECM‐producing population has recently been described that localizes to peripheral and subpleural regions of the IPF lung.^42^ In addition to fibroblasts, a *COL15A1* ^*+*^ endothelial cell population, that is generally observed underlying airways, has been reported in the distal IPF lung close to fibrotic foci and the area of bronchiolization.^31^ Phenotypic analysis of these cells needs to be performed, and their role in IPF is currently unknown. Further coculturing these cells with AT2 cells could elucidate their potential roles in modulating AT2 cells in IPF pathogenesis.

COPD encompasses a number of individual progressive lung diseases, including emphysema and chronic bronchitis, and is thought to affect 251 million people globally, with prevalence growing.^44^ COPD destroys the alveoli and leads to permanent enlargement of the respiratory airspaces, but disease etiology is unknown, although it is observed that smoking is the leading risk factor in disease initiation.^45^ Unlike interstitial lung diseases, obvious fibrosis is not observed in COPD. It is thought that continuous exposure to toxic chemicals initiate inflammatory‐oxidative stress, leading to damage of alveolar walls and the lung parenchyma, possibly through over‐activated oxidative metabolism. Antioxidants such as the haemoxygenase (HO)‐1 pathway may not be efficient in COPD, as reduced expression of HO‐1 has been observed in macrophages and bronchoalveolar lavage (BAL) fluid of COPD patients with a history of smoking.^46^ It has been postulated that dysfunction of AMs, coupled with bacterial persistence promotes COPD progression.^47^ scRNA‐seq analysis has identified aberrant basaloid cells within human COPD lungs, however they are observed far less frequently than in IPF, and their role in COPD pathogenesis remains to be validated.^31^ In addition to oxidative stress and immune dysfunction, structural changes to the lung may also play a part in the airflow limitation that characterizes COPD, such as the loss of CC10^+^ club cells in the human distal lung of COPD patients. It is unknown whether such changes are a direct result of cigarette smoke exposure or an indirect effect of persistent inflammation. The implication of inflammation within COPD could be modeled in a human‐specific manner, including the use of PCLS from COPD patients for functional and phenotypic characterization of immune cells. No single mechanism has been identified that can be responsible for the complex pathology of COPD, highlighting the importance of investigating interactions between different mechanisms. Another disease associated with severe inflammation is ARDS, a heterogeneous disease in which neutrophils infiltrate the lungs, where they migrate into the airways and express proinflammatory cytokines including TNFα and IL1β, causing hypoxemia, lung edema formation, and loss of pulmonary compliance.^48^, ^49^ ARDS results in breakdown of the alveolar‐capillary unit, affecting both epithelial and endothelial cells.^50^ ARDS could also benefit from human‐specific modeling, including the use of PCLS. In addition to cellular dysfunction, insufficient generation of alveoli can lead to disease. BPD is a chronic lung disease that affects newborns (usually premature) and infants. It arises almost exclusively in infants who have undergone mechanical positive pressure ventilation, suggesting that alveolar stretch and mechanical trauma play critical roles in BPD pathogenesis, although a combination of factors are potentially involved, and the cellular mechanisms underlying BPD development are not well understood.^51^ Due to lack of full growth and maturation of AT2 cells and alveoli, premature infants produce less surfactant, complicating ventilation strategies.^52^, ^53^ Furthermore, increased concentration of proinflammatory cytokines, including TNFα and IL1β, correlate with increased risk of BPD, while fibroblast conversion to invasive myofibroblasts is also observed.^54^ Both of these diseases would benefit from the establishment of in vitro models that replicate the histopathological aspects of diseases and are suitable for studying disease mechanisms and therapeutic interventions.

### 1.3 Lung in a dish: In vitro systems to model lung regeneration and disease

Desperate demands to find a suitable model to complement animal studies and provide more relevance to human biology led to development of 3D organoid cultures which are self‐organizing, multicellular structures formed from stem cells.^55^ The ability to establish and expand organoids from various human tissues including patients raises the possibility of using organoids in translational applications, such as in vitro disease modeling, personalized therapy and regenerative medicine as well as the establishment of organoid biobanks.^55^ Significant efforts and progress have been made in recent years within the field of lung organoid research. Until recently, research primarily involved the establishment and study of airway cell types that can be grown in both 2D air‐liquid interface (ALI) culture and 3D organoids, in part due to their general ease of culturing and increased availability of material through noninvasive bronchoscopic brushings or collection of bronchoalveolar lavage fluid. Recent advances in lung organoids reported the molecular requirements that enable adult human basal cells, embryonic lung tip progenitors, and pluripotent stem cells (hPSC) to expand and differentiate into multiple airway cell types either in 3D culture or when transplanted into injured mouse lung.^10^, ^18^, ^56^, ^57^, ^58^, ^59^ However, in vitro culturing of alveolar cell types both in human and mouse is challenging with suboptimal conditions, suggesting that there are still gaps in our knowledge in regard to human and mouse alveolar development, maintenance, and regeneration. Therefore, culture models containing alveolar cell types are severely lagging behind their airway counterparts.

#### 1.3.1 *Alveolar organoids*

The first human alveolar organoids from adult tissue were formed from AT2 cells isolated by a surface marker HTII‐280 and cocultured with MRC5 fibroblasts.^12^ Although forming spheres of cells that could be passaged at least once, indicating self‐renewal capacity of human AT2 cells, AT1 cells were not present, suggesting that MRC5 fibroblasts are not sufficient to fully support differentiation of AT2 cells. Cells were grown overnight in commercial media prior to sorting and plating, possibly leading to preferential selection of particular cell subsets. A later study utilized similar techniques to demonstrate that a subset of adult human HTII‐280^+^ AT2 cells expressing TM4SF1 were more capable of forming organoids.^2^ However, long‐term culture was not supported and morphology and cellular composition varied to the previous study, most likely due to differences in cell isolation strategies and culture conditions (Table. 1). As an alternative to adult stem cells, there have been increasing efforts in culturing alveolar cells as organoids derived from hPSCs. It was shown that alveolar spheroids could be produced through directed differentiation of hPSCs to anterior foregut endoderm, followed by an NKX2.1^+^ lung progenitor phase and induction of an SFTPC^+^ subset.^60^, ^63^ Building upon this, two separate studies generated improved protocols for induction of AT2 cells, and established alveolar organoids.^61^, ^62^ Both studies achieved induction of SFTPC^+^ AT2 cells without mesenchymal support, although long‐term culture of AT2 cells in these conditions was only reported in one study, possibly due to differences in cell derivation (Table 1).^61^, ^62^ Additionally, differentiation to AT1 cells could only be achieved by plating in 2D with serum‐containing medium.^64^ Temporal activation of Wnt signaling has been found to promote AT2 maturation.^62^ However, culture of hPSCs in Collagen I gels along with GSK3 inhibition using the small molecule CHIR99021 (CHIR) promoted proliferation and inhibited differentiation, whereas withdrawal of the inhibition induced multilineage maturation of proximal and distal fates.^66^ This finding could not be recapitulated using the Wnt ligand WNT3a, perhaps suggesting a method of action other than canonical Wnt signaling. This work contradicts previous studies, in which CHIR removal drove a proximal fate and continued presence led to AT2 cell fate, but may be due to differences in experimental set‐up.^59^, ^62^

**TABLE 1:** Current strategies for establishing human alveolar organoids from pluripotent stem cells, embryonic progenitors, or adult tissue

Method of isolation	Culture conditions	Passage	Cell types present	Significant findings	Reference
Adult AT2 cells					
Mechanical and enzymatic dissociation, followed by FACS using Epcam and HTII‐280 (epithelial and human AT2 cell markers, respectively)	MRC5 fibroblasts and ALI‐medium^65^	Passaged until passage 3	Some SFTPC^+^ AT2 cells	First time human AT2 cells were shown to be stem cells of the adult distal lung	12
Mechanical and enzymatic dissociation using a gentleMACS, followed by sorting using MACS for Epcam, HTII‐280, and TM4SF1 (for Wnt‐responsive “alveolar epithelial progenitors”)	MRC5 fibroblasts and MTEC Plus or SAGM medium (Lonza)	Not reported. Analyzed after 14‐21 days of primary culture	SFTPC^+^ AT2 cells and AQP5^+^ AT1 cells	TM4SF1 was used to isolate “alveolar epithelial progenitor” cells, which are proposed to be Wnt‐responsive and have increased regenerative capacity	2
Embryonic					
Tissue from human embryonic lungs of 5–20 postconceptional weeks (pcw) was enzymatically digested, and tips and stalks were dissected off and placed into matrigel for culture	Self‐renewal medium including 50 ng/mL recombinant human EGF, 100 ng/mL recombinant human Noggin, 100 ng/mL recombinant human FGF10, 100 ng/mL recombinant human FGF7, 3 mM CHIR99021 and 10 mM SB431542	Could be maintained in culture long‐term	SOX9^+^ and SOX2^+^ cells. Could be transplanted into NOD‐SCID immunocompromised mice, where they formed airway‐like cells. Could also be transplanted under the mouse kidney capsule to produce bronchiolar and alveolar cells	Identified key differences between mouse and human lung development, including the coexpression of SOX9 and SOX2 in human pseudoglandular distal tips	10
12‐week fetal lungs were mechanically and enzymatically dissociated, and lung buds were placed into matrigel to culture lung bud tip progenitor	Serum‐free basal medium with added factors, including 1X B27 supplement, 2 mM Glutamax, 1x Pennicillin‐Streptomycin, 0.05% Bovine Serum Albumin, FGF7, FGF10, BMP4, All‐trans retinoic acid, and CHIR‐99021	Not reported	SOX9^+^ and SOX2^+^ cells. Weak SFTPC staining and no protein staining for markers such as P63 or HOPX, consistent with human fetal epithelial bud tips prior to 16 wk gestation	Discovered that a combination of FGF7, CHIR‐99021, and RA were sufficient to maintain SOX9 expression (and SOX2 expression) in vitro	[57]
PSCs and iPSCs					
Human pluripotent stem cells were differentiated into lung epithelial cells via an NKX2.1^+^ “ventralized” anterior foregut endoderm stage	Cocultured with human fetal lung fibroblasts, plus step‐wise addition of factors including RA, CHIR99021, and BMP4 (for differentiation into NKX2.1+ cells), followed by FGF10, dexamethasone, 8‐Br‐cAMP, 3‐IBMX, and FGF7	Not reported	SFTPC^+^, SFTPB^+^, AQP5, NKX2.1	Identified carboxypeptidase M (CPM) as a surface marker of NKX2.1^+^ “ventralized” anterior foregut endoderm cells	60
NKX2.1^+^ ventralized anterior foregut endoderm cells were preconditioned using a treatment to mimic the microenvironment of distal tip cells (to induce SFTPC expression). CPM high cells were isolated using FACS, and plated in matrigel to establish alveolar organoids	FGF7, FGF10, Dexamathasone, 8‐Br‐cAMP, 3IBMX, CHIR‐99021, and SB431542	Fibroblast dependent = over 200 days. Fibroblast independent not disclosed	AT2 cells and AT1‐like cells	Used alveolar organoids as a drug toxicology model	61
Reporter lines of anterior foregut endoderm cells were sorted for NKX2.1 GFP^+^/SFTPC tdTomato^+^ expression, or CD47hi/CD26lo	FGF7, FGF10, Dexamathasone, 8‐Br‐cAMP, 3IBMX, CHIR‐99021, and SB431542 for increased SFTPC differentiation. Removing CHIR99021 from culture media for 1 week allowed cell maturation, followed by its subsequent addition for increased proliferation	Achieved serial passage without mesenchymal coculture	AT2 cells	Used CRISPR/Cas9 to correct a mutation within the SFTPB gene of AT2 cells in alveolar organoids	62
Human pluripotent stem cells were used to form NKX2.1^+^ ventral foregut spheroids, which were plated in matrigel	Serum‐free basal medium with added factors, including 1X B27 supplement, 2 mM Glutamax, 1x Penicillin–Streptomycin, 0.05% Bovine Serum Albumin, FGF7, All‐trans retinoic acid, and CHIR‐99021. CHIR‐99021 and RA were withdrawn for differentiation experiments	Survived for over 16 weeks in culture	Cells coexpressed NKX2.1 and SOX2, with peripheral budded regions also staining for SOX9 Small number of SCGB1A1^+^ cells and MUC5AC^+^ cells within interior of organoids. Following removal of CHIR‐99021 and RA, organoids displayed decreased SOX9 expression and expressed markers such as PDPN, HOPX, pro‐SFTPC, SFTPB, and ABCA3	Resulting organoids could be engrafted into the airways of immunocompromised mice, where they persisted for up to 6 wk, but basal cells or alveolar cells were not observed	58

In addition to studying cell regulation, organoids can also be used to model disease. Using hPSCs, organoids were produced using CRISPR/Cas9 that successfully modeled Hermansky‐Pudlak syndrome (HPS), a rare disorder in which mutations within three of the associated genes (HPS1, HPS2, or HPS4) result in the development of pulmonary fibrosis.^57^, ^67^ The resulting organoids displayed a fibrotic phenotype, with an enhanced number of mesenchymal cells and increased deposition of fibronectin and collagen. Patient‐derived organoids were also established. Building upon this, the cytokine IL‐11 was found to be upregulated in both fibrotic organoids and IPF patients, and was essential for the induction of fibrosis within mutant organoids.^68^ Such findings could one day lead to improved targeted therapies for disease treatment using pharmacological intervention. Another way that diseases such as IPF and BPD have been modeled is by culturing primary human fetal fibroblasts as 3D‐lung organoids using sodium alginate beads, where cell‐cell interactions could be monitored.^69^, ^70^ Future studies involving alveolar organoids will aim to increase our understanding of alveolar epithelial stem/progenitor cells and how they interact with their neighboring cells, in addition to improving disease modeling. Thus far, it has proven difficult to coculture human lung AT2 cells with multiple other cell types at once, such as mesenchymal, endothelial cells and immune cells, while still maintaining appropriate proliferation and differentiation, due to the cells’ differing culture requirements. However, increased understanding of how each individual cellular component is regulated may allow for improved coculture models. Furthermore, lung‐on‐a‐chip technology will enable analysis of multiple cell type interactions in microfluidic devices that will better recapitulate the complexity of the lung.

#### 1.3.2 *Precision‐cut lung slices*

PCLS recapitulate tissue‐specific features such as polarity and cellular architecture. Infusion of lung tissues with heated liquid agarose and subsequent solidification maintained alveolar structure when cutting, providing an advantage over traditional monolayer or organoid cell cultures.^71^, ^72^ PCLS are often utilized in toxicological and anatomical studies regarding contractility in lung diseases such as asthma and emphysema.^73^, ^74^ Immunological studies were also conducted in PCLS as they retain immune cell populations and functions.^75^, ^76^, ^77^, ^78^ More recently, individual live epithelial cells labeled by EPCAM and AT2 cells labeled by SP‐C have been traced in PCLS up to 64 hours during mouse alveologenesis using time‐lapse imaging analysis.^79^ However, one of the clear benefits of using PCLS is that human lung tissue, either from healthy donor or diseased lungs, can be employed, allowing for the study of cellular reactions in response to exogeneous factors or drugs while maintaining their anatomical structures and diverse cellular components. For example, in order to assess cellular behaviors of AT2 cells and activation of surrounding fibrosis in IPF lung tissues, human IPF‐derived PCLS were utilized. Inhibition of Notch signaling revealed an increase in mature SP‐B with a reduction of ECM staining in IPF‐PCLS that were treated with Notch inhibitor DAPT.^80^ Lipopolysaccharides (LPS), found ubiquitously in the environment but also in cigarette smoke, are implicated in acute lung injuries and chronic diseases such as COPD, and have a wide range of immune‐modulatory effects.^81^, ^82^ Treatment of human PCLS with LPS resulted in an increase in cytokines such as IL‐1β, while levels were reduced following exposure to dexamethasone, suggesting its potential use as an anti‐inflammatory treatment.^82^ Chemical‐induced immunotoxicity was assessed in human PCLS by analyzing tissue injury, viability, and the presence of inflammatory cytokines, where it was found that respiratory sensitizers such as HClpt increase TNF‐α and IL1‐α levels.^83^ Furthermore, gene transfer to human lung epithelium has been assessed using PCLS from macroscopically normal human lung tissues, where a LacZ‐expressing adenovirus reporter was instilled into bronchioles, and traced following 4 days in culture.^84^ The use of PCLS allowed visualization of β‐galactosidase in the lungs, providing the potential tissue‐relevant preclinical model of gene therapy. Despite the benefits, PCLS remain controversial, with some questioning how closely they recapitulate the in vivo tissue, in addition to their reliability during long‐term culture, an important consideration, particularly in applications such as drug testing of slowly metabolized chemicals.^85^ Furthermore, they are typically a static system, making analysis of breathing‐related diseases such as BPD difficult, although strategies have been developed that apply stretch to cultures, through methods such as suturing slices to a flexible membrane.^86^ Cell trafficking from the blood into the lungs, and vice versa, cannot be assessed, although future engineering efforts, particularly using technologies such as “organ‐on‐a‐chip” may be able to solve this limitation. Recruitable immune cells can also not be analyzed, and heterogeneity can exist in different regions even within a single lobe, although this could be rectified in part by sampling multiple regions. Donor variation is also an issue due to differences in genetic background, although the same problem arises in all models that utilize human tissue, and highlights the potential of personalized therapies. As tissue viability dramatically declines following isolation, improvements to cryopreservation efforts have allowed for immediate freezing and long‐term storage of tissue that can be used later in PCLS with no decline in cell viability and only slight decline in metabolic activity.^87^

#### 1.3.3 *Biology meets engineering*

Recapitulation of structural and cellular complexity of the lung encourages the use of bioengineering approaches to gain better microenvironmental control, and have led to the development of organ‐ or lung‐on‐a‐chip technologies. In addition to designing cell‐cell interactions, ‘on‐a‐chip’ technology has also provided the opportunity to better model tissue‐tissue and multiorgan interactions. One of their benefits is that mechanical forces, such as stretch and airflow can be implemented, and their effects on cell behavior monitored. This is an important consideration in creating more physiologically relevant cell models, particularly in the lung, as the cellular response of AT2 cells to the mechanical tension during alveolar development and regeneration of mouse lungs has been described.^88^, ^89^ Current examples using hPSCs or human alveolar epithelial cells have successfully recapitulated functional alveolar‐capillary networks and established a heart‐liver‐lung model in a closed‐loop system that was able to identify previously unknown cardiotoxicity of the chemotherapeutic drug bleomycin through analyzing multitissue crosstalk.^90^, ^91^ Further implementation of stretch and breathing motions may result in improved cell differentiation or maturation of implemented alveolar cell types. Primary human lung alveolar epithelial and endothelial cells were recently cultured on a “breathing” lung‐on‐a‐chip platform.^92^ This system not only allowed a breathing motion but also possessed a mechanism for passive medium exchange, better recapitulating the in vivo lung environment. Biological validation revealed that AT2 and AT1 cells could be maintained in culture on the chip for a number of days, thus overcoming the challenge of using microfluidic devices for primary cells, which are usually damaged or stressed due to their fragile nature. Recently, the inhalation system has been coupled with a lung‐on‐a‐chip device containing human airway cells.^93^, ^94^ This system has the benefit of using microfluidics and breathing motions along with the exposure of smoke, possibly toxins or viruses, within a contained environment, and allows for controlled dosing of the tested stimulus, and could be used for analysis of the exposure effect on the human lung epithelium. It would be interesting to connect “airway chip” to “alveolar chip,” which would increase the applicability of the model to the human lung. Lung‐on‐a‐chip systems have now evolved to include 3D lung organoids, named organoid‐on‐a‐chip, which contain all of the key cell types, rather than the established lung cell lines traditionally used, providing a better platform with microphysiological features.^95^, ^96^

For more tissue‐relevant modeling, decellularization of the lung, in which cells are removed while the ECM is retained, has been attempted.^97^ This is an attractive alternative therapy strategy, where the decellularized lung can act as a scaffold upon which the patient's own stem/progenitor cells could be attached. As immune‐related cells will also be removed, immune rejection upon transplantation should be overcome. Furthermore, decellularized lung could also act as an ex vivo model of disease.^98^ The cellular phenotype of rat AT2 cells have been altered by losing lamellar bodies and gaining a flattened morphology when delivered to decellularized human lung, suggesting transdifferentiation to AT1‐like cells, but the effect was slower when cells were cultured on scaffolds from acellular human amniotic basement membranes, highlighting the importance of region‐specific ECM.^99^, ^100^ Besides decellularized lung, synthetic scaffolds have also been investigated, and lung organoids can be cultured on such scaffolds to improve cell survival and engraftment upon transplantation, and have been tested in the mouse epididymal fat pad, where pseudostratified airway‐like structures were observed after introduction of hPSC‐derived lung organoids.^101^ Such scaffolds can also result in more mature cell types produced in vivo, although alveolar cell types were not present, and it remains to be discovered whether synthetic scaffolds can induce human alveolar cell maturation.^101^

#### 1.3.4 *Current drawbacks*

Human AT2 cells are characterized by expression of a number of different markers, including SFTPC, SFTPB, ABCA3, and HTII‐280 (Figure 2B). HTII‐280, a monoclonal antibody that recognizes the apical membrane of human AT2 cells, has up until recently been the only surface marker used to specifically isolate human AT2 cells from primary human tissue.^2^, ^12^, ^102^ Multiple studies have found that HTII‐280^+^ AT2 cells are enriched within the healthy epithelium of the human distal lung. Flow‐cytometry analysis of human AT2 cells that were isolated by mechanical and enzymatic dissociation of clinical parenchymal lung tissue samples demonstrated that HTII‐280^+^ AT2 cells represent between 70% and 90% of epithelial cells (Figure 2C), and are significantly reduced in IPF patient tissue samples (Figure 3C). This can depend on tissue quality and method of isolation, with fragile AT1 cells often becoming damaged during processing.^2^, ^12^, ^41^ However, it is important to note that although the majority of AT2 cells express HTII‐280, HTII‐280^−^ AT2 cells are observed even in healthy human lungs (Figure 2B). Furthermore, HTII‐280 is a less useful marker for subculture of AT2 cells due to loss of expression during culture.^67^ Alternative isolation strategies are therefore currently under investigation. Lysotracker, a fluorescent dye that labels acidic components within lysosomes, has been shown to successfully label the lamellar bodies of AT2 cells, allowing for both live cell imaging and selection of high‐expressing cells using fluorescence‐activated cell sorting (FACS).^61^, ^67^, ^80^ More recently, NaPi2b, a sodium phosphate cotransporter that is highly expressed on the surface of AT2 cells, has been shown to isolate a HTII‐280^+^ equivalent population of SFTPC^+^ cells.^67^ This same marker was also used for passaging hPSC‐derived organoids, where it was found to be more useful for subculturing AT2 cells than HTII‐280.^67^ Furthermore, MHCII has been previously used to isolate AT2 cells from mouse lungs, and the MHCII antigens HLA‐DR and HLA‐DP are expressed on the surface of human AT2 cells.^103^, ^104^, ^105^ Advances in scRNA‐seq techniques may begin to identify more reliable surface markers for isolation of human AT2 cells, and establishment of reporter cell lines will be helpful in subculturing of AT2 cells.^61^, ^62^ Furthermore, establishment of collaborative online tools such as LungMAP (www.lungmap.net) and the Human Lung Cell Atlas will aid researchers in establishing improved isolation strategies.^106^ It is important to consider that reliance on single lineage marker strategies can be unreliable, particularly when multiple cell types may express the same marker (Table 2). Furthermore, disease conditions may affect gene expression profiles, altering normal marker expression, such as the case in IPF with the observance of cells expressing both airway and alveolar marker genes.

**TABLE 2:** Lineage markers for diverse human lung epithelial cell types

Human epithelial cell type	Established lineage markers	Suggested lineage markers	References
Alveolar Type II (AT2)	ABCA3, HTII‐280, LAMP3, LPCAT1, SFTPC, SFTPA, SFTPB, SFTPD	*TM4SF1, MUC1	1, 2, 10, 12, 57, 61, 62, 64, 102
Alveolar Type I (AT1)	AGER, AQP5, CAV‐1, HOPX, HTI‐56, PDPN	IGFBP2, CLIC5	1, 10, 107
Basal	KRT5, *KRT14, *NGFR, TP63, PDPN	KRT15, KRT17, DAPL1	1, 18, 108
Secretory club	*PLUNC, SCGB1A1, *SCGB3A1, *SCGB3A2	CCKAR, CYP2F2	1, 101
Ciliated	Acetylated tubulin, β3‐tubulin, FOXJ1	TUBB1	1, 101
Goblet	MUC5AC, MUC5B, SPDEF		101

*Note:* Suggested lineage markers have not yet been fully validated.

a Expressed in a subset of cells.

A current issue with establishment of in vitro human alveolar model systems is the lack of defined culture media for their growth and maintenance. This has impeded studies to analyze precise regulatory mechanisms and cellular requirements for supporting alveolar stem/progenitor cells and maintaining their differentiated lineages and multiple different cell types including stromal cells, immune cells, and endothelial cells. Long‐term expansion of adult lung alveolar organoids with sustained functional lineages, as well as efficient isolation and expansion of cells from limited material for clinical applications have also been challenging. This issue can be solved in part by deriving organoids from hPSCs, which can be expanded on a larger scale from less starting material. However, there are increased safety concerns with using hPSCs, particularly in autologous transplantation, due to the possible presence of undifferentiated cells with the potential of tumorigenic events.^109^ Epigenetic memory following induction is also a concern, and cells will have to be of an assured quality.^110^ Such issues need to be considered and addressed before being used in the clinic, and patients monitored post‐transplantation. It has also been noted that culture differences between separate laboratories can lead to considerable heterogeneity within a single induced‐hPSC cell line, while interpatient heterogeneity also exists.^111^, ^112^, ^113^ Another consideration that needs to be made for both adult‐ and hPSC‐derived organoids is the method in which the cells are cultured, with most relying on matrigel, a mixture of undefined ECM components secreted by Engelbreth‐Holm‐Swarm mouse sarcoma cells.^114^ As a result, matrigel is not approved for use in humans. The undefined nature of matrigel may also result in issues such as inhibition of cell maturity or differentiation. Therefore, this has led to increased efforts in producing defined, synthetic hydrogels, such as those utilized for intestinal organoids to aid in colonic wound healing.^115^

### 1.4 Translational applications and stem cell therapies

A progression from using organoid models to gain better understanding of cellular behavior during regeneration and disease is to apply this knowledge in developing improved translational treatments. CRISPR/Cas9 technology has been used in lung organoids to successfully model various lung diseases and apply gene correction.^56^, ^62^, ^68^ An HPS2 gene mutation in induced‐hPSC organoids derived from patients of HPS was successfully corrected using CRISPR/Cas9, with restoration of transcript levels of the protein‐trafficking gene *AP3B1*.^67^ A separate study achieved gene correction of a mutant form of the SFTPB gene in induced‐hPSC‐derived alveolar organoids isolated from an infant with neonatal respiratory distress.^62^ Correction resulted in restored *SFTPB* transcript levels, visible lamellar bodies, and reconstitution of mature SFTPB protein within AT2 cells. Furthermore, a more recent study has achieved reversal of SFTPB protein deficiency by introducing wild‐type SFTPB gene in patient‐derived induced‐hPSC organoids through lentiviral transduction.^116^ However, whether such cells could be used to achieve disease reversal in patients remains to be elucidated. For lung cell therapy, the feasible mode of cell introduction to the distal alveoli remains challenging. Furthermore, for CRISPR/Cas9 technology, it is important to fully understand the safety concerns regarding potential off‐target mutations, a topic which has conflicting views in regard to the significance of such effects.^117^, ^118^

For stem cell therapies, there are two main ways in which transplanted cells may result in benefits to the patient; cell‐engraftment or so‐called bystander effects. Cell‐engraftment involves the incorporation of cells, for example, epithelial stem/progenitor cells into the damaged epithelium of a tissue, where they can replace the damaged epithelium by expansion and/or differentiation. In this way, any improvements to tissue integrity or disease outcome are a direct effect of transplanted cells. On the other hand, bystander effects involve the modulation of the host tissue through secreted cues. This often involves paracrine signaling, resulting in immune modulation and promotion of epithelial and endothelial repair, or deposition of ECM.^119^ Transplantation of mesenchymal stromal cells (MSCs) to aid repair in lung diseases such as COPD, ARDS, and BPD has been investigated.^120^, ^121^, ^122^ Human bone marrow‐derived MSCs reduced fibrosis and inflammation in mouse model of COPD, as observed by reduction of IL‐1β and TNF‐α.^123^ Treatment of an ARDS rat‐model with human umbilical cord MSCs increased survival and oxygenation, and reduced IL‐6 and TNF‐α levels.^124^ Allogenic transplantation of bone‐marrow‐derived MSCs has been tested in patients suffering with chronic lung allograft dysfunction after lung transplantation.^121^ Although demonstrating the feasibility and relative safety of the approach, the potential of MSCs to differentiate into profibrotic cells remains a concern.

The possibility of using a patient's own epithelial cells without gene correction has been explored. A rare population of human basal cells expressing SOX9 were enriched in 2D culture following bronchoscopic brushings from Bronchiectasis patients, and instilled back into the individual patients’ lung lobes, leading to thinner bronchial walls and improved pulmonary function.^125^ The direct contribution of delivered cells and the long‐term effects of this procedure are currently unknown, and reproducibility needs to be proven. Such findings highlight the increasing “stem cell hype” that is present within both the media and wider scientific community. This can lead to over‐emphasis of findings, which can have a host of consequences including misleading the public, reduction in methodical scientific approaches, and even potentially harmful premature clinical use.^126^ The importance to carefully validate scientific and clinical findings, as well as increase study sizes, is therefore evident.

## 2 CONCLUSION AND FUTURE PERSPECTIVES

In vitro human alveolar models provide a new powerful platform to investigate cellular behavior and activity of human lung alveolar stem/progenitor cells, cell‐cell crosstalk, host‐pathogen interactions, as well as to conduct drug screenings and toxicity assays. Despite these advances, improved model systems to recapitulate complexity of in vivo human lungs are still required. Additionally, recent scRNA‐seq technologies have elucidated cellular and molecular heterogeneity and diversity within the human lung, with the observance of “aberrant” cell types arising during disease states that are not present within healthy lungs. Additional single‐cell “omics” approaches with spatial information are becoming available. Such cells await further characterization and phenotypic analysis to assess their roles in disease pathogenesis, but their identification may hold the key to better understanding lung diseases and repair mechanisms. Use of human lung‐derived cells in autologous transplantation may promise a future treatment of lung disease, although a number of hurdles, including the efficacy of transplantation and the method of administration, need to be overcome before these translational efforts will be realized in the clinic. Engrafting cells onto cellular scaffolds prior to transplantation may assist in increasing cell survivability and maturation, while production of injectable synthetic hydrogels could improve cell delivery. Most importantly, common standard principles for tissue acquisition and processing are required. Human alveolar lung models are invaluable tools to address these questions, and may one day lead to therapeutic regeneration of the human lung.

## CONFLICT OF INTEREST

The authors declared no potential conflicts of interest.

## AUTHOR CONTRIBUTIONS

K.V.E.: designed experiments, interpreted the data, performed experiments and data analysis, and wrote the manuscript; J.‐H.L.: designed experiments, interpreted the data, and wrote the manuscript.

## Data Availability

Data sharing is not applicable to this article as no new data were created or analyzed in this study.
